# Three plasma metabolite signatures for diagnosing high altitude pulmonary edema

**DOI:** 10.1038/srep15126

**Published:** 2015-10-13

**Authors:** Li Guo, Guangguo Tan, Ping Liu, Huijie Li, Lulu Tang, Lan Huang, Qian Ren

**Affiliations:** 1 Department of Cardiology, Xinqiao Hospital, Third Military Medical University, Chongqing 400042, China; 2 Department of Pharmaceutical Analysis, School of Pharmacy, Fourth Military Medical University, Xi’an 710032, China; 3 Department of outpatient, No. 22 Hospital of PLA, Geermu 816000, China; 4 State key laboratory of Medical Genetics and school of life sciences, central south university, changsha, 430013, China; 5 Department of Medical Teaching, Daping Hospital, Third Military Medical University, Chongqing 400042, China

## Abstract

High-altitude pulmonary edema (HAPE) is a potentially fatal condition, occurring at altitudes greater than 3,000 m and affecting rapidly ascending, non-acclimatized healthy individuals. However, the lack of biomarkers for this disease still constitutes a bottleneck in the clinical diagnosis. Here, ultra-high performance liquid chromatography coupled with Q-TOF mass spectrometry was applied to study plasma metabolite profiling from 57 HAPE and 57 control subjects. 14 differential plasma metabolites responsible for the discrimination between the two groups from discovery set (35 HAPE subjects and 35 healthy controls) were identified. Furthermore, 3 of the 14 metabolites (C8-ceramide, sphingosine and glutamine) were selected as candidate diagnostic biomarkers for HAPE using metabolic pathway impact analysis. The feasibility of using the combination of these three biomarkers for HAPE was evaluated, where the area under the receiver operating characteristic curve (AUC) was 0.981 and 0.942 in the discovery set and the validation set (22 HAPE subjects and 22 healthy controls), respectively. Taken together, these results suggested that this composite plasma metabolite signature may be used in HAPE diagnosis, especially after further investigation and verification with larger samples.

High altitude pulmonary edema (HAPE) is a life threatening clinical condition, mostly occurring in non-acclimatized healthy individuals who rapidly ascend to high altitude (above 3000 m)^1^. It is the major cause of death related to high altitude exposure. Currently, HAPE diagnosis mainly relies on patient interviews, physician’s examination, X-ray radiograph and computed tomography (CT) of the chest, and magnetic resonance imaging (MRI)^2^, as there are lack of objective laboratory-based tests. Current diagnostic methods are highly dependent on the clinician experience, which usually results in underdiagnosis, delayed diagnosis, and misdiagnosis due to the high heterogeneity of clinical symptoms. Therefore, the identification of metabolite biomarkers for HAPE would be of great clinical value in laboratory-based diagnosis of HAPE and understanding the pathophysiology of the disease.

Metabonomics is a top-down systems biology approach whereby metabolic responses to disease or treatment are analyzed and modeled^3^. Hence, metabonomics represents an excellent developing prospect for capturing diseases specific metabolic signatures as possible biomarkers^4^. Metabolite biomarkers have been successfully applied in the discrimination or diagnosis of various diseases such as cancer^5^, neurodegenerative diseases^6^, cardiovascular disease^7^, diabetes^8^, and so on. Previously, there was a report on identifying the molecular alterations associated with HAPE by ^1^H NMR- based metabonomics approach^9^, which not only provided valuable clues in dissecting the mechanisms of HAPE, but also exemplified the ability of metabonomics to identify diagnostic biomarkers for HAPE from clinical samples. Given that ^1^H NMR analytical technology cannot provide complete coverage of the human metabonome due to the diverse physicochemical properties of metabolites and the relatively low sensitivity of ^1^H NMR, the metabolite variations and the disturbances of metabolic pathways of HAPE are still far from complete. It is meaningful to apply complementary metabonomic platforms such as mass spectrometry to identify novel biomarkers of HAPE.

In this study, we therefore applied metabonomic method based on ultra-high performance liquid chromatography (UHPLC) coupled with Q-TOF mass spectrometry to profile metabolites in plasma samples from 57 HAPE subjects and 57 healthy controls. One of the purposes was to identify the differential plasma metabolites in HAPE patients relative to healthy controls. The other purpose was to optimize a simplified metabolite signature for HAPE diagnosis.

## Materials and Methods

### Participants

The study protocol was approved by the Human Ethics Committee of the Third Military Medical University, and written informed consent was obtained from all study volunteers prior to participation. All procedures involving the human subjects were carried out in accordance with the recommendations of the Helsinki Declaration.

A total of 57 HAPE subjects were enrolled as cases for the study, including 49 men and 8 women with a mean age of 34.79 ± 5.84 years, who developed the disease after traveling from the lowlands to Golmud district (altitude 2,780–4,500 m) in Qinghai, China. All subjects were recruited between March 2013 and December 2014 from the 22nd Hospital of the Chinese People’s Liberation Army, which is the largest hospital located in the city of Golmud in Qinghai. This hospital is the primary treatment center for individuals suffering from high altitude disease in this region, which is considered as a “checkpoint” to Tibet by many travelers. The HAPE patients enrolled in this study did not receive any medication therapy prior to sample collection. Fasting blood samples were collected from HAPE patients. The diagnosis of HAPE was based on standard criteria^10^ including cough, dyspnea, cyanosis at rest, absence of infection, the presence of pulmonary rales and cyanosis. In all cases, HAPE was confirmed by chest radiographic findings of infiltrates consistent with pulmonary edema. The control group consisted of 49 men and 8 women with a mean age of 34.66 ± 6.08 years. These individuals were selected according to a 1:1 case-matching scheme using the variables sex, age, blood pressure, BMI and method of ascent. All control subjects were non-natives of a high altitude environment who had not developed any symptoms or signs of HAPE or related illness after exposure to high altitude within 7 days. Blood samples from healthy volunteers were obtained under fasting conditions.

In total, the 57 HAPE patients and 57 healthy controls were enrolled into this study and then were divided into a discovery set and a validation set. The discovery set, composed of 35 HAPE subjects and 35 healthy controls, was used to identify plasma diagnostic markers for HAPE; the remaining subjects were used to establish the validation set to independently validate the diagnostic generalizability of these biomarkers. The detailed demographic and clinical data of the participants are presented in Table 1. Any patients with previous history of cardiopulmonary diseases and other metabolic diseases such as diabetes, hypertension, obesity and heart disease, as identified by self-reported medical history or full examination carried out after HAPE recovery, were excluded from study participation.

### Chemicals and reagents

HPLC-grade Methanol and acetonitrile (ACN) were purchased from Merk (Darmstadt, Germany). Formic acid was obtained from Fluka (Buchs, Switzerland). Sphingosine and palmitoylcarnitine were purchased from Acros Organics (NewJersey, USA). Glutamine, methionine, hypoxanthine, inosine, valine and isoleucine were obtained from Shanghai Jingchun Reagent Co. Ultrapure water was prepared with a Milli-Q water purification system (Millipore, Bedford, MA, USA).

### Sample preparation

Fasting venous blood (with EDTA as an anticoagulant) was obtained from all the above-mentioned individuals. The plasma was separated immediately by centrifugation (3000 × g, 10 min). The harvested plasma samples were stored at −80 °C, and transported to Shanghai for further experiments. Prior to the analysis, a volume of 400 μL of methanol was added to 100 μL of plasma. After vigorous shaking for 1 min and incubation on ice for 10 min, the mixture was centrifuged at 14,000 × g for 15 min at 4 °C to precipitate the protein. All the supernatant was removed (without removing any particles left at the bottom of the vial). The supernatant was evaporated to dryness with a gentle nitrogen stream. The dry residue was reconstituted in 100 μL of ACN/water (7:3, v/v), then centrifuged again at 14,000 × g for 10 min at 4 °C.

As part of the system conditioning and quality control (QC) process, a pooled QC sample was prepared by mixing equal volumes (100 μL) from each of the 114 samples as they were being aliquoted for analysis. It was processed as real samples and then was inserted through the analytical run at intervals of 8–13 real samples to be analyzed eleven times. The QC samples were sufficiently spread out through the whole run as to ensure its validity.

### UHPLC-Q-TOFMS analysis

UHPLC analysis was performed on Agilent 1290 Infinity LC system (Agilent, Germany). Chromatographic separation was carried out at 40 °C on an ACQUITY UPLC BEH C_18_ column (2.1 mm × 100 mm, 1.7 μm, Waters, Milford, MA). The column oven was set at 40 °C. The mobile phase consisted of 0.1% formic acid (A) and ACN modified with 0.1% formic acid (B), using a gradient elution of 5%B at 0–2 min, 5%–95% B at 2–13 min, 95% B at 13–15 min. The total run time was 20 min including equilibration. The flow rate was 350 μL/min and the injection volume was 4 μL.

An Agilent 6530 Accurate-Mass Quadrupole Time-of-Flight (Q-TOF) mass spectrometer (Agilent, USA) was used in the study. The Q-TOF mass spectrometer was operated in electrospray ionization source (ESI) positive ion mode with a capillary voltage of 3.5 kV, drying gas flow of 11 L/min, and a gas temperature of 350 °C. The nebulizer pressure was set at 45 psig. The fragmentor voltage was set at 120 V and skimmer voltage was set at 60 V. All analyses were acquired using a mixture of 10 mM purine (*m/z* 121.0508) and 2 mM hexakis phosphazine (*m/z* 922.0097) as internal standards to ensure mass accuracy and reproducibility. Data were collected in centroid mode and the mass range was set at *m/z* 50–1000 using extended dynamic range. Potential biomarkers were analyzed by MS/MS in the Q-TOF. Nitrogen was used as the collision gas. MS/MS analysis was performed on the mass spectrometer set at different collision energy of 10−50 eV according to the stability of each metabolites. MS spectra were collected at 2 spectra/s, and MS/MS spectra were collected at 0.5 spectra/s, with a medium isolation window (~4 *m/z*). A negative ion scan was only employed when metabolite identification was carried out.

### Data Handling

The raw data in instrument specific format (.d) were converted to common data format (.mzData) files using a conversion software program (file converter program available in Agilent MassHunter Qualitative software), in which the isotope interferences were eliminated. The program XCMS (version, 1.40.0) (http://masspec.scripps.edu/xcms/xcms.php) was used for nonlinear alignment of the data in the time domain and automatic integration and extraction of the peak intensities^11^. XCMS parameters were default settings (major default parameters: profmethod = bin; method = matchedFilter; step = 0.1) except for the following: full width at half maximum (FWHM) = 8, bandwidth (bw) = 10 and snthresh = 5, due to narrower peaks obtained by the use of the column packed with 1.7 μm particles. The variables presenting in at least 80% of either group were extracted^12^, and the variables with a retention time less than 0.5 min (near to the dead time) were excluded due to a high degree of ion suppression that they suffered^13^. Variables with less than 30% relative standard deviation (RSD) in QC samples^14^ were then retained for further multivariate data analysis because they were considered stable enough for prolonged UHPLC–Q-TOFMS analysis. For each chromatogram, the intensity of each ion was normalized to the total ion intensity, in order to partially compensate for the concentration bias of metabolites between samples and to obtain the relative intensity of metabolites. The resulting three-dimensional matrix, including retention time and *m/z* pairs (variable indices), sample names (observations), and normalized ion intensities (variables), was exported to multivariate data analysis.

The normalized data was introduced to SIMCA-P V11.0 (Umetrics, Sweden) for principal component analysis (PCA) and partial least squares discriminant analysis (PLS-DA) after mean-centering and pareto scaling, a technique that increased the importance of low abundance ions without significant amplification of noise. The quality of the models was evaluated with the relevant *R*^2^ and *Q*^2^ as well discussed elsewhere^15^. T-test was performed in succession to reveal the statistical differences for the variables between healthy and HPAE individuals.

## Results

### Plasma metabolic profiling by UHPLC-MS

The separation conditions of plasma on UHPLC-MS system were optimized in terms of peak shape and reproducibility. The representative chromatograms of plasma metabolomes in ESI positive mode are shown in Supplementary Fig. S1. The stability of the analytical method is very important to obtain valid metabonomic data. To validate the system performance during the analysis of real samples, a pooled QC sample was applied^16^, which was a representative “mean” sample including all the analytes during the analysis. The QC sample was processed as real samples and then was inserted amongst the real sample queue to be analyzed eleven times. PCA results of the QC sample demonstrated that the peak areas deviation was less than 2 SD, indicating that the data from the UHPLC-MS were statistically acceptable (supplemental Fig. S2). In addition, it was found that the variation and of the retention times are 0.02–0.06 min for metabolites of interest in QC samples, and the relative standard derivations (RSD) for peak areas of metabolites of interest are 4.2%–13.1% in QC samples (see Table 2 for data). All the results demonstrated the robustness of the method. This confirms that the significant differences observed between the two groups by multivariate statistical analysis were more likely to be a result of genuine subtle changes in metabolites, rather than products of artifacts arising from technical errors.

### Multivariate statistical analysis of metabolites

**Figure 1:**
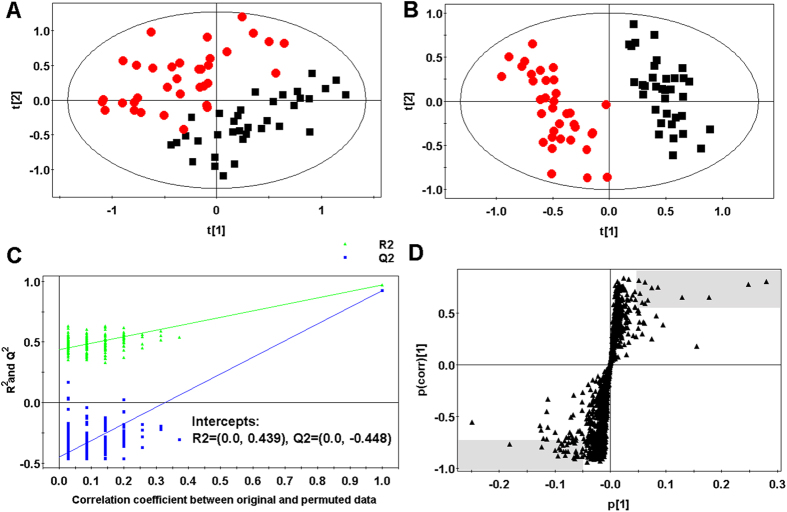
Multivariate data analysis. (**A**) PCA score map derived from UHPLC-Q-TOFMS spectra concerning healthy (■) and HAPE (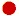) individuals. (**B**) PLS-DA score map derived from UHPLC-Q-TOFMS spectra concerning healthy (■) and HAPE (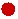) individuals. (**C**) Validation plot obtained from 99 permutation tests. (**D**) S-plot of the PLS-DA model.

The normalized data sets contained 1218 ions. To determine whether the metabolite fingerprints in plasma differed between the healthy and HAPE subjects, we first evaluated separation between healthy and HAPE subjects using unsupervised principal component analysis (PCA). The obvious separation was achieved between HAPE group and healthy group (R^2 ^= 0.75) (Fig. 1A). To further search ion peaks that can discriminate between the two groups, the supervised PLS-DA model was established in that it was more focused on the actual class discriminating variation compared to the unsupervised PCA model. A clear separation between healthy group and HAPE group was observed in the PLS-DA score plot by the first two components (Fig. 1B) (R^2 ^= 0.97, Q^2 ^= 0.93). To validate the model, permutation tests with 99 iterations were further performed. These permutation tests compared the goodness of fit of the original model with the goodness of fit of randomly permuted models. As shown in Fig. 1C, the validation plot indicates that the original model is valid. The criteria for validity are as follows: all the permuted R^2^ (cum) and Q^2^ (cum) values to the left are lower than the original point to the right, and the blue regression line of the Q^2^ (cum) points has a negative intercept^17^,^18^.

### Identification of differential plasma metabolites in HAPE

Metabolites were carefully screened before being approved as potential biomarkers. First, significant original variables were extracted from the S-plot, which is a covariance-correlation-based procedure, and thus the risk of false positives in metabolite selection was reduced^19^. The S-plot (Fig. 1D), derived from the first component of the combined model, explains most of the variables in data set, in which the ions furthest away from the origin contribute significantly to the clustering of the two groups and may be regarded as potential biomarkers (in two shaded areas of Fig. 1D). Next, the variable importance for projection (VIP) reflecting the importance of variables has been applied to filter the important metabolites in the model. The most important 30 variables were first selected according to their VIP value. Furthermore, the fold change of the relative intensity from the differential metabolite between the two groups was set as 1.5. Unpaired Student’s t-tests were performed as the final testing procedure, and the critical p-value was set to 0.05 for significantly differential variables. Following the criterion above, 14 metabolite ions were selected as potential biomarkers related to HAPE.

**Figure 2:**
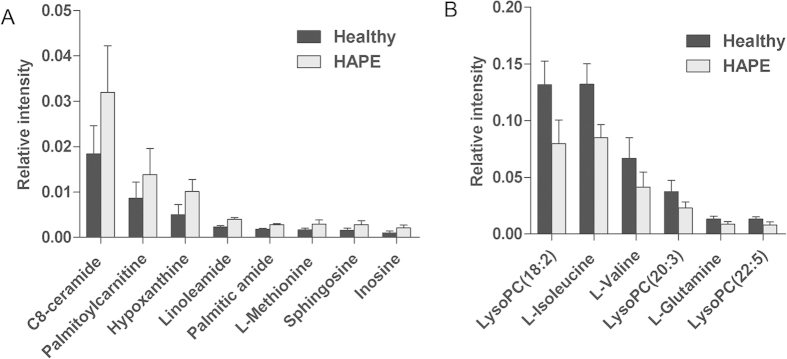
HAPE subjects possess increased/decreased metabolites. (**A**) The relative signal intensities of the increased metabolites in HAPE subjects. (**B**) The relative signal intensities of the decreased metabolites in HAPE subjects. Data are expressed as mean ± S.D.

The detailed method for the compound identification was described in the author previous work^20^. In brief, the corresponding quasi-molecular ion peak was found according to the accurate mass and retention time in the extracted ion chromatogram (EIC), and then the most probable molecular formula were calculated by Agilent MassHunter software. Fig. S3 A and S3 B show the EIC and MS spectrum of a typical ion whose *m/z* is 400.3420. Then, MS/MS analysis of *m/z* 400.3420 in plasma was performed using UHPLC-Q-TOFMS in the same chromatographic and mass spectrometric conditions (Fig. S3 C). With its fragmentation information and the freely accessible databases such as HMDB (http://www.hmdb.ca) and METLIN (http://metlin.scripps.edu), the major fragment ions *m/z* 341.2685, 144.1016, 85.0290 and 60.0813 represent the fragments of [C_20_H_37_O_4_]^+^, [C_7_H_14_NO_2_]^+^, [C_4_H_5_O_2_]^+^ and [C_3_H_9_N]^+^, respectively. Therefore, the *m/z* 400.3420 was identified as palmitoylcarnitine according to the elemental composition, retention time and fragmentation information. Finally, the MS/MS spectrum of the commercial standard palmitoylcarnitine was used to confirm the identified compound. Other biomarkers have been similarly identified and are listed in Table 2 and the structures and MS/MS spectra of the metabolites are presented in Supporting Information Fig. S4. Among these metabolites, the high level of C8-ceramide, palmitoylcarnitine, hypoxanthine, linoleamide, palmitic amide, methionine, sphingosine and inosine, and the low level of isoleucine, valine, glutamine, lysoPC(18:2), lysoPC(20:3) and lysoPC(22:5) were observed in HAPE subjects relative to healthy controls. (Fig. 2)

### Identification of a simplified HAPE metabolite signature

**Figure 3:**
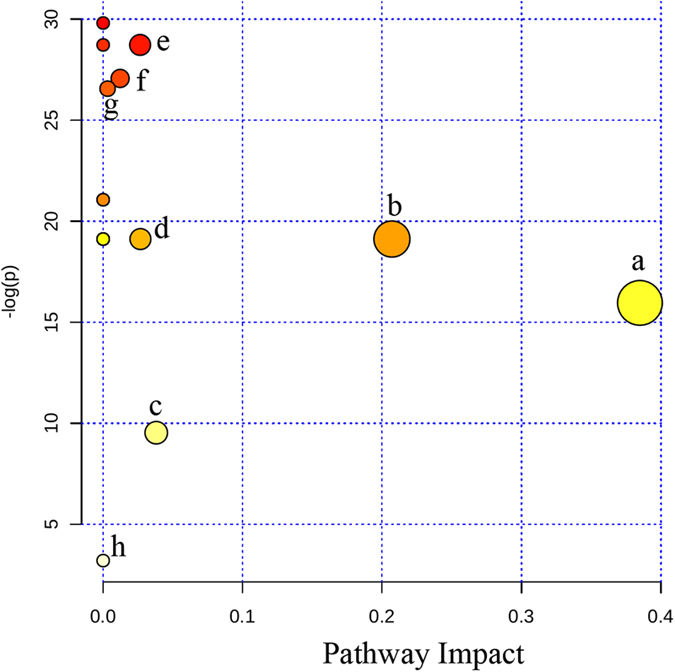
The pathway impact of HAPE on plasma metabolites with MetaboAnalyst 3.0. (**a**) sphingolipid metabolism; (**b**) alanine, aspartate and glutamate metabolism, (**c**) methionine metabolism; (**d**) D-Glutamine and D-glutamate metabolism; (**e**) valine, leucine and isoleucine biosynthesis; (**f**) purine metabolism; (**g**) phospholipid metabolism; (**h**) fatty acid metabolism.

14 candidate plasma biomarkers of HAPE were identified in the above analysis. However, diagnosis based on quantification of so many metabolites would not be economical and convenient in clinical practice. It would be more practical in diagnosing HAPE to identify a simplified plasma metabolite signature. Therefore, the 14 differential metabolites were used as candidates for selection of a simplified HAPE metabolite signature. It is well-known that changes in more important positions of a network will trigger a more severe impact on the pathway than changes occurring in marginal or relatively isolated positions^21^. The metabolic pathway impact analysis with MetaboAnalyst 3.0 revealed that these differential metabolites are important for the organism response to HAPE and are responsible for multiple pathways^22^,^23^. Therefore, it was used to optimize a plasma metabolite signature for HAPE. The metabolic networks are directed graph as Fig. 3. It was revealed that the identified metabolites are responsible for sphingolipid metabolism, alanine, aspartate and glutamate metabolism, methionine metabolism, D-Glutamine and D-glutamate metabolism, valine, leucine and isoleucine biosynthesis, purine metabolism, fatty acid metabolism and phospholipid metabolism. The impact-value threshold calculated from pathway topology analysis was set to 0.10 24, and two unique pathways including sphingolipid metabolism and alanine, aspartate and glutamate metabolism was filtered out as potential targets pathway for HAPE. Potential metabolite signature was then identified from the two metabolic pathways. Among the 14 differential metabolites, C8-ceramide and sphingosine belong to sphingolipid metabolism, and glutamine belongs to alanine, aspartate and glutamate metabolism. Therefore, it was speculated that these three metabolites should yield the higher predictive power for future diagnostic applications. The relative concentrations of these three plasma metabolite biomarkers for HAPE are presented in Fig. 2.

**Figure 4:**
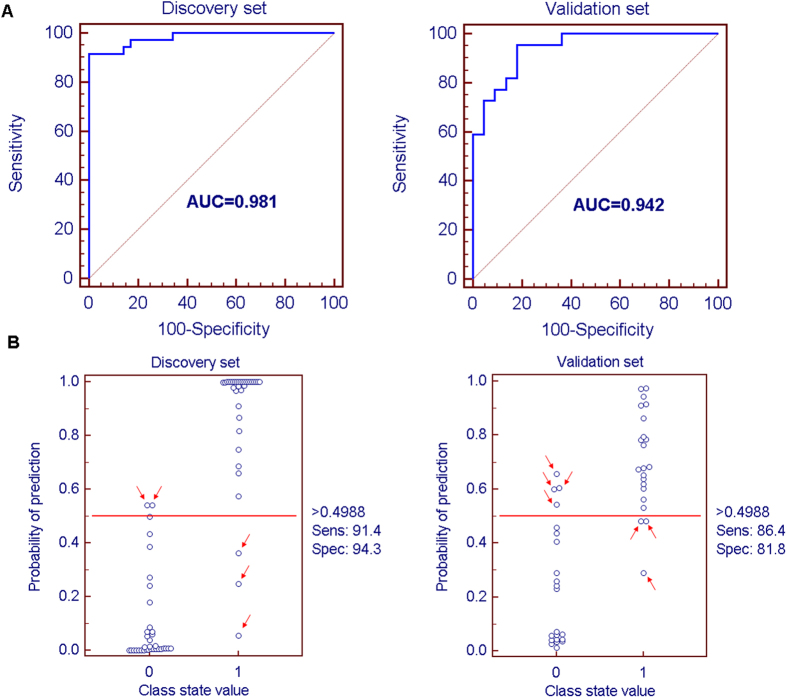
ROC curves based on the binary logistic regression model by the combination of three plasma metabolites (C8-ceramide, sphingosine and glutamine), and their prediction plots based on the optimal cutoff value from ROC curves. (**A**) The HAPE samples from the discovery set were applied to construct a binary logistic regression model based on the combination of plasma C8-ceramide, sphingosine and glutamine, and the ROC curves of the discovery set ((**A**), left) and validation set ((**A**), right) were obtained from the above established prediction model. (**B**) The optimal cutoff value with the highest sensitivity and specificity in the ROC curves of the training set was obtained (0.4988) and applied to evaluate the prediction capacity (92.8% for discovery set ((**B**), left) and 84.1% for validation set ((**B**), right)) of the current model, where 0 and 1 on the x axis represent healthy controls and HAPE patients, respectively, and blue circle represent samples.

To further validate the potential diagnostic effectiveness of the simplified metabolite signature, the ROC-curve was plotted using relative intensities of metabolites. The three representative metabolites including C8-ceramide, sphingosine and glutamine were selected as a panel of candidate markers. Logistic regression was used to combine the three variables into a multivariable. The prediction model is as follows: P = 1/[1 + exp(−(−2.39 + 204.16 × (C8-ceramide) + 3015.73 × (sphingosine) − 829.96 × (glutamine)))]. The results indicated that a panel of three metabolites generated an AUC of 0.981 with a sensitivity of 91.43% and a specificity of 94.29% and 0.942 with a sensitivity of 86.36% and a specificity of 81.82 for the discovery and validation sets, respectively (Fig. 4A). According to the highest prediction sensitivity (91.43%) and specificity (94.29%) of the ROC curves on the discovery set, an optimal cutoff value of 0.4988 was obtained. Based on this cutoff value, it was found that 65 out of 70 samples (92.8%) in the discovery set as well as 37 out of 44 samples (84.1%) in validation set could be accurately predicted (Fig. 4B). This finding indicated that this simplified plasma metabolite signature was a “good” classifier of HAPE patients and healthy controls.

## Discussion

HAPE is a severely life-threatening acute mountain sickness that endangers the lives of people climbing or migrating to high altitudes. Currently, the lack of disease biomarkers constitutes a bottleneck in the clinical diagnosis of HAPE. Here, using UHPLC-Q-TOFMS-based plasma metabonomic approach, 14 potential biomarkers related to HAPE was identified. Compared to previous ^1^H NMR-based study, UHPLC-Q-TOFMS-based metabolomic approach provided larger coverage of HAPE-related metabonome including sphingolipid, phospholipids, fatty acid amides and several amino acids. Based on metabolic pathway impact analysis, a metabolite signature consisting of three plasma metabolite biomarkers C8-ceramide, sphingosine and glutamine was further identified as an effective diagnostic pattern, yielding an AUC of 0.981 in the discovery set and 0.942 in the validation set. Our results suggest that this metabolite signature may be helpful in the development of objective laboratory-based diagnostic tools for HAPE.

**Figure 5:**
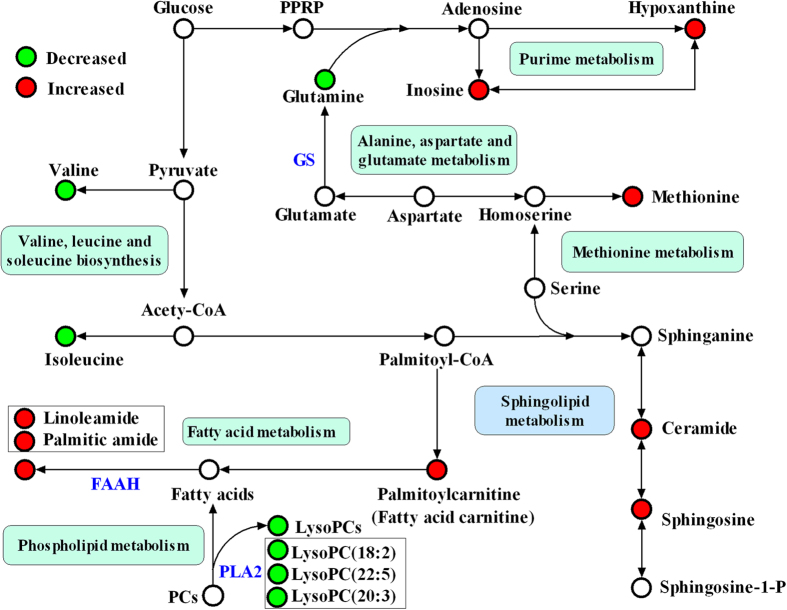
Schematic overview of the disturbed metabolic pathways associated with HAPE. The metabolites are shown in color: red represents increased metabolites, green represents decreased metabolites and the open circles represent no detected or changed metabolites in our experiment. PRPP, phosphoribosyl pyrophosphate; GS, Glutamine synthetase

To understand the underlying molecular functions of these plasma metabolite biomarkers, metabolic pathway analysis was conducted. The 14 metabolites were found to be primarily involved in (a) sphingolipid metabolism (b) alanine, aspartate and glutamate metabolism, (c) methionine metabolism, (d) D-Glutamine and D-glutamate metabolism, (e) valine, leucine and isoleucine biosynthesis, (f) purine metabolism, (g) fatty acid metabolism, (g) phospholipid metabolism and (h) fatty acid metabolism. By relating the metabolic pathways, the metabolic network of HAPE-related potential biomarkers was constructed (Fig. 5). The disturbed metabolic pathways are discussed in detail below.

The significantly higher levels of C-8 ceramide and sphingosine were observed in HAPE subjects relative to healthy controls, suggesting the sphingolipid metabolism is upregulated in HAPE subjects. It was reported that ceramide-challenged pulmonary endothelial cells exhibit decreased barrier function, independent of apoptosis^25^,^26^, which may contribute to lung inflammation and pulmonary edema^27^. A recent study also reported that exogenous sphingosine-1-phosphate boosted acclimatization in rats exposed to acute hypobaric hypoxia^28^. In addition, the level of palmitoylcarnitine was significantly increased in HAPE subjects relative to healthy controls, which illustrated that HAPE facilitated the process of sphingolipid biosynthesis. These consistent set of findings suggested that reestablishing the sphingolipid homeostasis was an important drug target for improving physiological acclimatization of subjects venturing into high altitude.

The low level of glutamine, a key metabolite in the pathway of alanine, aspartate and glutamate metabolism and D-Glutamine and D-glutamate metabolism, was observed in HAPE subjects. Since the biosynthesis of glutamine depends on glutamine synthetase, we speculated that the decrease in activities of glutamine synthetase should be a reason for the decrease of glutamine. In agreement with this presumption, several studies have consistently reported that the activity of glutamine synthetase was decreased in animal model exposed to high altitude (4000 m)^29^.

A significantly higher level of methionine was observed in HAPE subjects relative to healthy controls, suggesting the methionine metabolism pathway is perturbed in HAPE subjects. Although the metabolic mechanism is not yet well defined, it is plausible that the metabolic disorder results from dysregulation of proteolysis, oxidative catabolism, and gluconeogenesis^30^. Further study on its underlying mechanisms is underway in our laboratory.

The levels of inosine and hypoxanthine were significantly increased in HAPE subjects relative to healthy controls. Inosine and hypoxanthine are the products of adenosine metabolic degradation. It was previously reported that adenosine was released by hypoxic canine lung tissue and the levels of inosine and hypoxanthine showed sustained significant increases^31^, which is consistent with our results. In addition, an increased purine metabolism flux induced by acute systematic hypoxia has also been observed in a recently reported LC-MS study^32^. These data suggests that perturbed purine metabolism is implicated in HAPE.

The low levels of valine and isoleucine, correlated with valine, leucine and isoleucine biosynthesis, were observed in HAPE subjects. Valine and isoleucine, two branched-chain amino acids, may be an important alternative energy substrate. It seems that the reduction in ATP production due to the inhibition of citrate cycle induced by the hypobaric hypoxia of high altitude could lead to the utilization of branched-chain amino acids as energy compensation. Branched-chain amino acids have been suggested as a useful supplementation in the treatment of lung disease^33^ and in trekking at high altitude^34^. These results together with our findings suggest that HAPE is associated with disturbances in branched-chain amino acids metabolism.

Fatty acid amides (FAMs) are a group of endogenous lipid signaling molecules found in the brain and blood of mammals. Linoleamide and palmitic amide were FAMs and a significantly higher levels of them were observed in HAPE subjects relative to healthy controls. Although the possible role of linoleamide and palmitic amide in HAPE could not be directly elucidated, the physiological functions of the other FAM such as endocannabinoid anandamide could give an indirect clue to them. It was previously reported that endocannabinoid anandamide could mediate hypoxic pulmonary vasoconstriction via fatty acid amide hydrolase (FAAH)-dependent metabolites and hypoxia could cause elevated anandamide in the lung^35^. Meanwhile, exaggerated hypoxic pulmonary vasoconstriction is one of the pathological features of HAPE^1^. In line with these previous reports, we speculated that linoleamide and palmitic amide may also be an important mediator of hypoxic pulmonary vasoconstriction and be involved in the generation of pulmonary hypertension. The mechanism of action of the two FAMs still carried out in our laboratory.

Lysophosphatidylcholines (LysoPCs) including LysoPC(18:2), LysoPC(20:3) and LysoPC(22:5) were obviously decreased in plasma from HAPE subjects relative to healthy controls, suggesting the phospholipid metabolism is implicated in stroke patients. LysoPC is an important signaling molecule with diverse biological functions and can mediate many cell signaling pathways in monocytes/macrophages and specific receptors^36^,^37^, so that it participates in inflammatory response. The regulation of phospholipid metabolites may have important implications in inflammation response following HAPE induced-by hypobaric hypoxia.

## Conclusion

In conclusion, an UHPLC−Q-TOFMS based metabonomic approach has been developed to profile HAPE-related metabolic changing in plasma. Fourteen potential biomarkers have been identified as being primarily involved in sphingolipid metabolism, alanine, aspartate and glutamate metabolism, methionine metabolism, D-Glutamine and D-glutamate metabolism, valine, leucine and isoleucine biosynthesis, purine metabolism, fatty acid metabolism and phospholipid metabolism. Using metabolic pathway impact analysis and metabolite enrichment analysis, we identified a panel of plasma metabolite biomarkers relating to HAPE, of which the combination of plasma C8-ceramide, sphingosine and glutamine could discriminate HAPE patients from healthy controls with high accuracy. It suggested that this composite urinary metabolite signature may have diagnostic and/or prognostic values for HAPE, which deserve to be further investigated in larger populations with accurately characterized patients and to explore their corresponding mechanisms related to HAPE.

## Additional Information

**How to cite this article**: Guo, L. *et al.* Three plasma metabolite signatures for diagnosing high altitude pulmonary edema. *Sci. Rep.* **5**, 15126; doi: 10.1038/srep15126 (2015).

## Supplementary Material

Supplementary Information

## Figures and Tables

**Table 1:** Demographic and clinical details of recruited subjects

	Discovery Set			Validation set		
	Control	HAPE	p value	Control	HAPE	p value
sample size	35	35	−	22	22	−
sex (M/F)	31/4	31/4	−	18/4	18/4	−
Age, year	34.81 ± 6.03	35.00 ± 5.75	0.88	34.42 ± 6.29	34.45 ± 6.10	0.98
Method of ascent	by train	by train	−	by train	by train	−
Systolic blood pressure, mm Hg	119.57 ± 3.75	120.56 ± 3.51	0.25	120.76 ± 3.86	119.91 ± 3.60	0.45
Diastolic blood pressure, mm Hg	78.27 ± 4.06	78.15 ± 4.00	0.89	77.87± 4.22	78.22 ± 3.74	0.77
Body mass index, kg/m2	22.45 ± 2.29	22.27 ± 2.12	0.73	22.39 ± 2.45	22.58 ± 2.19	0.78
arterial oxygen saturation, %	92.34 ± 3.89	77.48 ± 5.99	<0.0001	92.39 ± 3.39	76.54 ± 5.67	<0.0001
Pulse rate, rate/min	81.47 ± 5.82	94.95 ± 10.82	<0.0001	81.47 ± 5.82	94.95 ± 10.82	<0.0001

**Table 2:** Potential biomarkers and their metabolic pathways.

No.	*m/z*	t_R_(min)	Formula	Metabolite	Ratio^a^	VIP^b^	Trend^c^	Related pathway	%RSD^d^
1	147.0761	0.81	C_5_H_10_N_2_O_3_	Glutamine^e^	0.67	2.65	↓^*^	Alanine, aspartate and glutamate metabolism	12.7
2	150.0581	0.97	C_5_H_11_NO_2_S	Methionine^e^	1.75	1.41	↑^*^	Methionine metabolism	10.3
3	118.0860	1.10	C_5_H_11_NO_2_	Valine^e^	0.62	6.07	↓^*^	Valine, leucine and isoleucine biosynthesis	8.3
4	137.0456	1.14	C_5_H_4_N_4_O	Hypoxanthine^e^	2.02	2.97	↑^*^	Purine metabolism	7.9
5	269.0875	1.28	C_10_H_12_N_4_O_5_	Inosine^e^	2.14	1.39	↑^*^	Purine metabolism	13.1
6	132.1008	1.31	C_6_H_13_NO_2_	Isoleucine^e^	0.64	9.75	↓^*^	Valine, leucine and isoleucine biosynthesis	4.2
7	300.2887	9.54	C_18_H_37_NO_2_	Sphingosine^e^	1.81	1.41	↑^*^	Sphingolipid metabolism	8.9
8	400.3420	10.46	C_23_H_45_NO_4_	Palmitoylcarnitine^e^	1.60	2.39	↑^*^	Sphingolipid metabolism	7.5
9	520.3379	10.62	C_26_H_50_NO_7_P	LysoPC(18:2)^f^	0.60	9.73	↓^*^	Phospholipid metabolism	6.7
10	426.3574	10.66	C_26_H_51_NO_3_	C8−ceramide^f^	1.74	4.46	↑^*^	Sphingolipid metabolism	4.8
11	280.2631	10.83	C_18_H_33_NO	Linoleamide^f^	1.76	1.98	↑^*^	Fatty acid metabolism	10.3
12	570.3546	11.00	C_30_H_52_NO_7_P	LysoPC(22:5)^f^	0.60	3.01	↓^*^	Phospholipid metabolism	9.5
13	546.3549	11.14	C_28_H_52_NO_7_P	LysoPC(20:3)^f^	0.61	4.81	↓^*^	Phospholipid metabolism	5.4
14	256.2638	13.03	C_16_H_33_NO	Palmitic amide^f^	1.59	1.50	↑^*^	Fatty acid metabolism	10.8

^a^The ratio of relative amounts of HAPE group to control group.

^b^Variable Importance in Projection.

^c^Change trend compared with control group. (↑): up-regulated. (↓): down-regulated.

^d^Variation of the biomarker concentrations in QC samples expressed as relative standard deviation (%RSD).

^e^Metabolites validated with standard sample.

^f^Metabolites putatively annotated. *p value < 0.01.
