# Supplementary material for: Three plasma metabolite signatures for diagnosing high altitude pulmonary edema
Source: Sci Rep. 2015 Oct 13;5:15126. doi: 10.1038/srep15126 (PMC4602305; doi:10.1038/srep15126)
Supplement: Supplementary Information [file srep15126-s1.pdf]

Supplementary data

Three plasma metabolite signatures for diagnosing high altitude pulmonary edema

Li Guo[†,‖], Guangguo Tan*,[‡,‖],Ping Liu[§], Huijie Li[†], Lulu Tang[⊥], Lan Huang*[,†], Qian Ren[1,¶]

[†]Department of Cardiology, Xinqiao Hospital, Third Military Medical University, Chongqing 400042, China

[‡]Department of Pharmaceutical Analysis, School of Pharmacy, Fourth Military Medical University, Xi'an 710032, China

[§]Department of outpatient, No. 22 Hospital of PLA, Geermu 816000, China

[⊥]State key laboratory of Medical Genetics and school of life sciences, central south university, changsha, 430013, China

[¶]Department of Medical Teaching, Daping Hospital, Third Military Medical University, Chongqing 400042, China

[‖]Authors with equal contribution to the research.

[1] Corresponding Author: E-mail: renqian777@126.com; Tel.: +86-23-6875-7203; Fax: +86-23-6875-7203 (Ren Q). E-mail: lanhuang126@126.com (Huang L), E-mail: guangguotan@gmail.com; Tel.: +86-29-8477-6827 (Tan GG)

Supplementary data

Figure S1. Representative chromatogram of the HPAE patient's plasma in ESI positive mode based on UHPLC-Q-TOFMS. (A) Representative total ion current (TIC) chromatogram. (B) The extracted ion chromatogram (EIC) of potential biomarkers.

Figure S2. Quality control (QC) plots of eleven randomly repeated runs of UHPLC–MS analysis by principle component analysis using component 1 and 2. Peak area deviation could be evaluated by distribution of the runs. X-axis: run order; Y-axis: standard deviation. (A) QC plot for the first component from UHPLC–MS data; (B) QC plot for the second component from UHPLC–MS data.

Figure S3. Identification of a selected marker (*m/z* 400.3420). (A) The extracted ion chromatogram (EIC) of *m/z* 400.3420. (B) The MS spectra of *m/z* 400.3420. (C) The MS/MS spectra of *m/z* 400.3420; the MS/MS fragmentation pattern of *m/z* 400.3420 was acquired with collision energy at 15 eV.

Figure S4. Structures and MS/MS spectra of representative metabolites. (A) Glutamine, (B)Methionine, (C)Valine, (D)Hypoxanthine, (E)Inosine, (F) Isoleucine, (G) Sphingosine, (H) Palmitoylcarnitine, (I) LysoPC(18:2), (J) C8-ceramide, (K) Linoleamide, (L) LysoPC(22:5), (M) LysoPC(20:3) and (N) Palmitic amide.

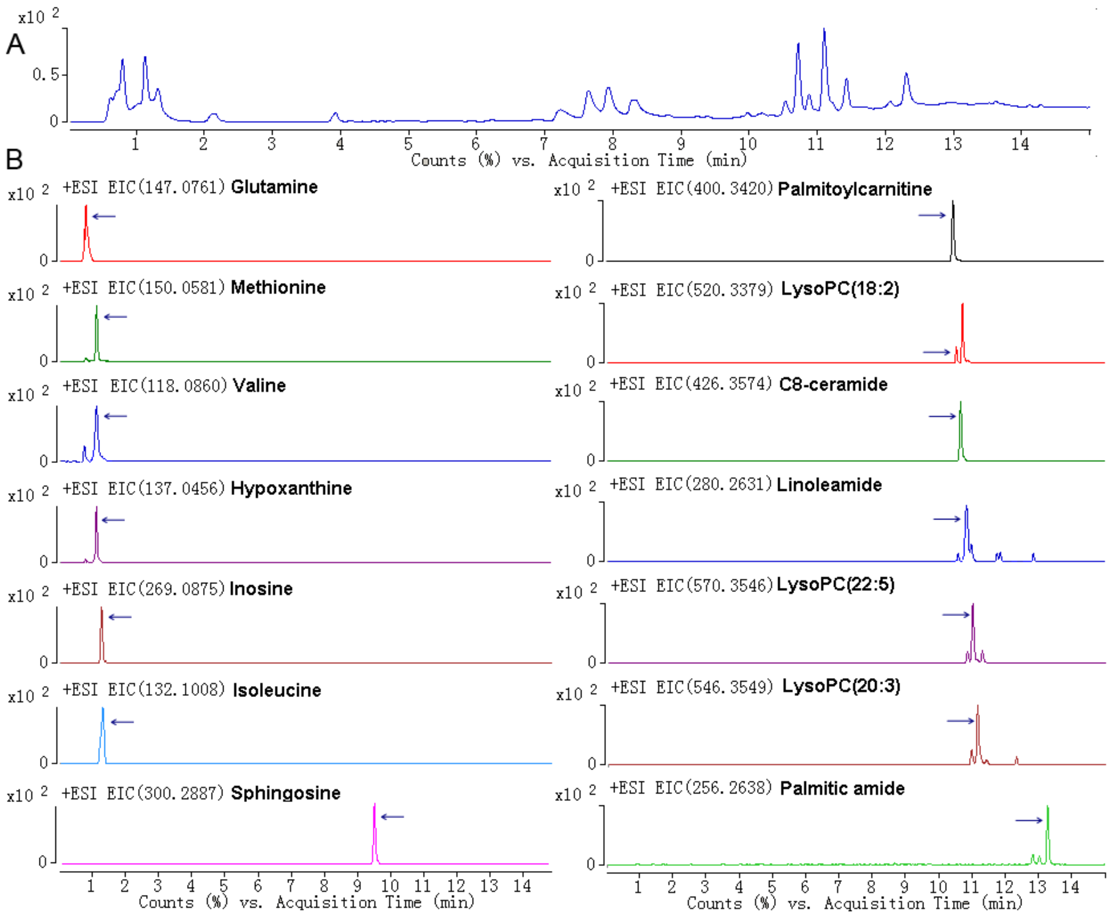

Figure S1. Representative chromatogram of the HPAE patient's plasma in ESI positive mode based on UHPLC-Q-TOFMS. (A) Representative total ion current (TIC) chromatogram. (B) The extracted ion chromatogram (EIC) of potential biomarkers.

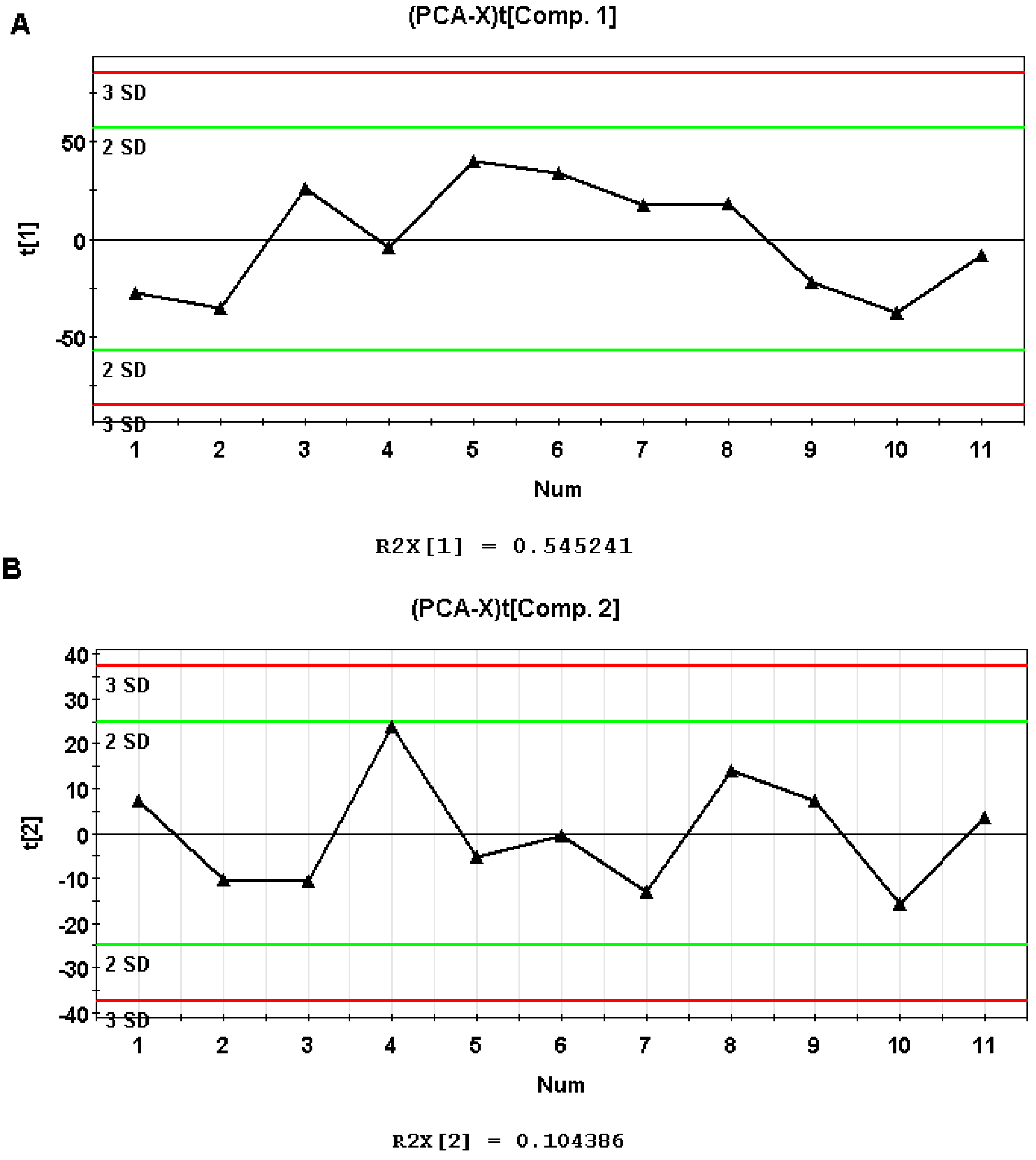

Figure S2. Quality control (QC) plots of eleven randomly repeated runs of UHPLC–MS analysis by principle component analysis using component 1 and 2. Peak area deviation could be evaluated by distribution of the runs. X-axis: run order; Y-axis: standard deviation. (A) QC plot for the first component from UHPLC–MS data; (B) QC plot for the second component from UHPLC–MS data.

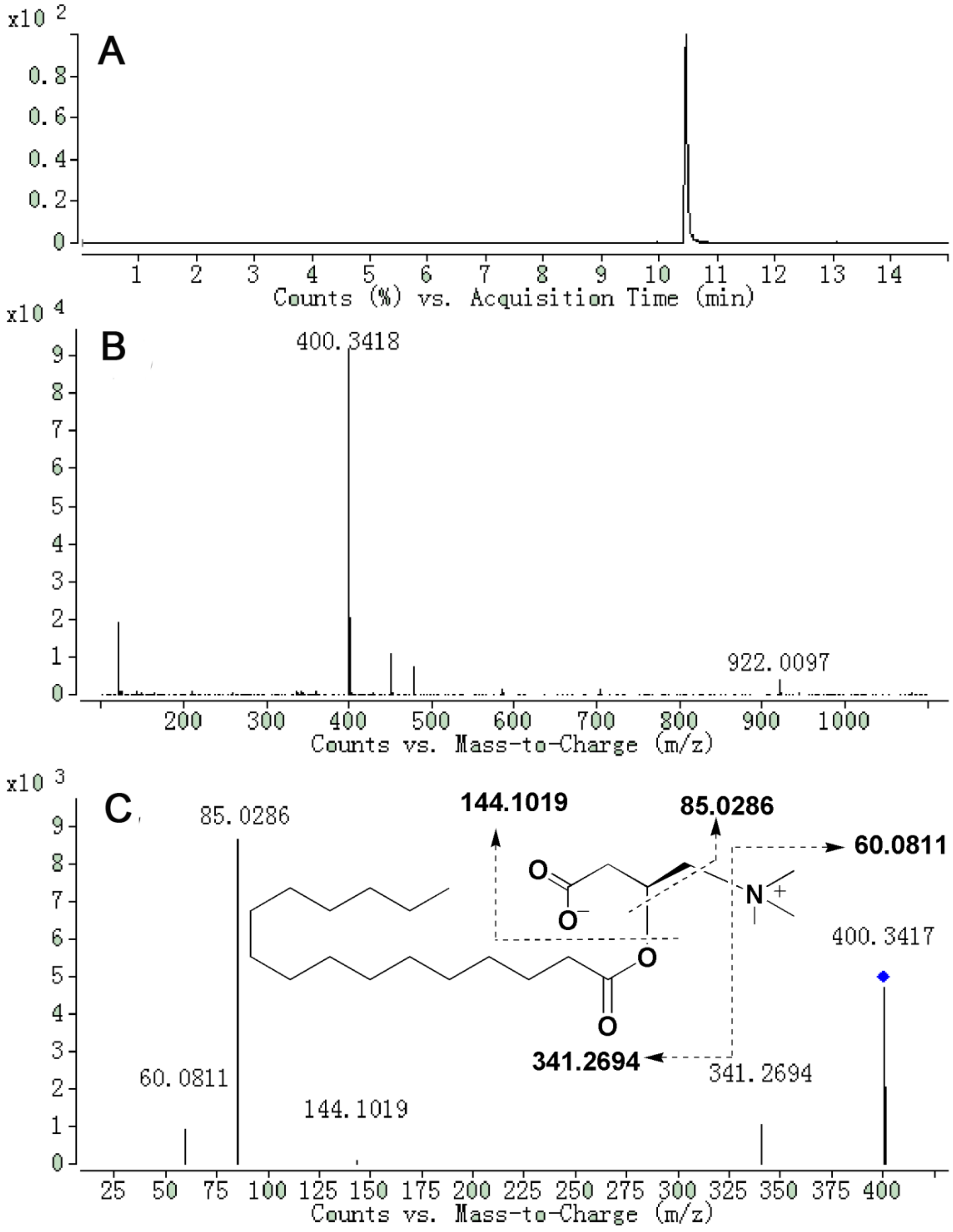

Figure S3. Identification of a selected marker (*m/z* 400.3420). (A) The extracted ion chromatogram (EIC) of *m/z* 400.3420. (B) The MS spectra of *m/z* 400.3420. (C) The MS/MS spectra of *m/z* 400.3420; the MS/MS fragmentation pattern of *m/z* 400.3420 was acquired with collision energy at 15 eV.

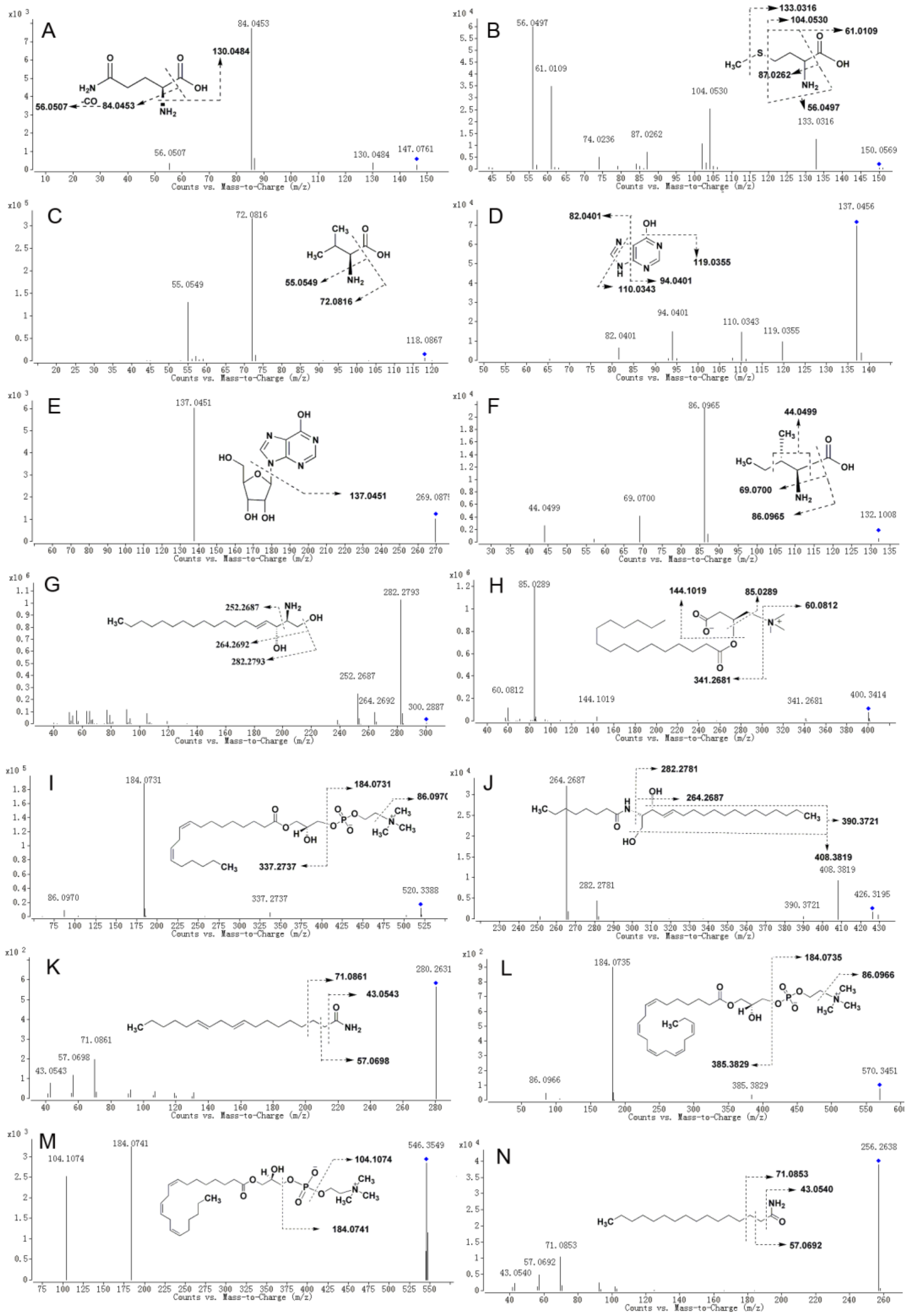

Figure S4. Structures and MS/MS spectra of representative metabolites. (A) Glutamine, (B)Methionine, (C)Valine, (D)Hypoxanthine, (E)Inosine, (F) Isoleucine, (G) Sphingosine, (H) Palmitoylcarnitine, (I) LysoPC(18:2), (J) C8-ceramide, (K) Linoleamide, (L) LysoPC(22:5), (M) LysoPC(20:3) and (N) Palmitic amide.
